# Proton Pump Inhibitor Intake neither Predisposes to Spontaneous Bacterial Peritonitis or Other Infections nor Increases Mortality in Patients with Cirrhosis and Ascites

**DOI:** 10.1371/journal.pone.0110503

**Published:** 2014-11-04

**Authors:** Mattias Mandorfer, Simona Bota, Philipp Schwabl, Theresa Bucsics, Nikolaus Pfisterer, Christian Summereder, Michael Hagmann, Alexander Blacky, Arnulf Ferlitsch, Wolfgang Sieghart, Michael Trauner, Markus Peck-Radosavljevic, Thomas Reiberger

**Affiliations:** 1 Division of Gastroenterology and Hepatology, Department of Internal Medicine III, Medical University of Vienna, Vienna, Austria; 2 Section for Medical Statistics, Center for Medical Statistics, Informatics, and Intelligent Systems, Medical University of Vienna, Vienna, Austria; 3 Clinical Institute of Hospital Hygiene, Vienna General Hospital, Vienna, Austria; 4 Vienna Hepatic Hemodynamic Lab, Division of Gastroenterology and Hepatology, Department of Internal Medicine III, Medical University of Vienna, Vienna, Austria; University of Modena & Reggio Emilia, Italy

## Abstract

**Background and Aim:**

The aim of this study was to assess the impact of proton pump inhibitor (PPI) intake on the development of spontaneous bacterial peritonitis (SBP) or other infections, as well as on mortality, in a thoroughly documented cohort of patients with cirrhosis and ascites.

**Patients and Methods:**

We performed a retrospective analysis of follow-up data from 607 consecutive patients with cirrhosis undergoing their first paracentesis at a tertiary center. A binary logistic regression model investigating the association between PPI intake and SBP at the first paracentesis was calculated. Competing risk analyses and Cox models were used to investigate the effect of PPIs on the cumulative incidence of SBP or other infections and transplant-free survival, respectively. Adjustments were made for age, hepatocellular carcinoma, history of variceal bleeding, varices and model of end-stage liver disease score.

**Results:**

Eighty-six percent of patients were receiving PPIs. After adjusting for potential confounding factors, PPI intake was neither associated with increased SBP prevalence at the first paracentesis (odds ratio (OR):1.11,95% confidence interval (95%CI):0.6–2.06; *P* = 0.731) nor cumulative incidence of SBP (subdistribution hazard ratio (SHR): 1.38; 95%CI:0.63–3.01; *P* = 0.42) and SBP or other infections (SHR:1.71; 95%CI:0.85–3.44; *P* = 0.13) during follow-up. Moreover, PPI intake had no impact on transplant-free survival in both the overall cohort (hazard ratio (HR):0.973,95%CI:0.719–1.317; *P* = 0.859) as well as in the subgroups of patients without SBP (HR:1.01,95%CI:0.72–1.42; *P* = 0.971) and without SBP or other infections at the first paracentesis (HR:0.944,95%CI:0.668–1.334; *P* = 0.742).

**Conclusions:**

The proportion of cirrhotic patients with PPI intake was higher than in previous reports, suggesting that PPI indications were interpreted liberally. In our cohort with a particularly high prevalence of PPI intake, we observed no association between PPIs and SBP or other infections, as well as mortality. Thus, the severity of liver disease and other factors, rather than PPI treatment *per se* may predispose for infectious complications.

## Introduction

Cirrhosis, which accounts for 1.8% of all deaths in Europe [1], is the 12^th^ leading cause of death in the United States, though a recent report suggests even this rank to be an underestimation [2].

According to a prognostic model proposed by D′Amico and co-workers [3], the occurrence of varices initiates the second stage of cirrhosis, the third stage is defined by the development of ascites and variceal hemorrhage initiates the fourth stage. The occurrence of bacterial infections, which delineates an additional fifth stage of cirrhosis termed the “critically ill” patient with cirrhosis [4], as it increases mortality of patients with decompensated cirrhosis up to four-fold. Thirty percent of patients die within 1 month and another 30% die during the first year after onset of infection [4]. These bacterial infections predominately occur in decompensated patients with advanced cirrhosis who typically have ascites. Spontaneous bacterial peritonitis (SBP) is the most common infection among patients with cirrhosis [5] and a consequence of quantitative and qualitative changes in gut microbiota, increased intestinal permeability and bacterial translocation [6]. In addition immunologic impairments observed in patients with advanced cirrhosis may play a role [5]. Small intestinal bacterial overgrowth (SIBO), a quantitative change of the gut microbiota, has been found to be associated with SBP development [7] and Chang and co-workers observed higher rates of SIBO among patients with a history of SBP [8]. Moreover, an association between SIBO and the presence of bacterial DNA in the peripheral blood of cirrhotic patients has been observed [9]. Impaired small intestinal motility [8], portal hypertension [10] and acid-suppressive therapy, such as proton pump inhibitors (PPIs) [11], have been reported as factors contributing to SIBO in patients with cirrhosis.

Several studies have observed an association between PPI intake and SBP development [12]–[18] and this relationship has recently been confirmed by a meta-analysis [19]. However, this association was not observed in all cohorts [16], [20], as demonstrated by one of the few prospective studies on the association between PPI intake and SBP development [20]. In fact, Kwon and co-workers [12] reported increased mortality after SBP development among patients with PPI intake, while other studies have observed a lack of effect on mortality [13], [20]. Moreover, several major limitations related to the study design as well as the consideration of potential confounding factors substantially limit the conclusions drawn from previous studies and the meta-analysis based on their results.

The aim of this study was to assess the impact of PPI intake on (i) the development of SBP or other infections, as well as (ii) on mortality, in a large, thoroughly documented cohort of patients with cirrhosis and ascites.

## Patients and Methods

### Study design

A total of 607 previously investigated [21] consecutive patients with cirrhosis who underwent their first paracentesis at the Medical University of Vienna between 2006 and 2011 were included in this retrospective study. Patients were followed up until 2011. Patients with other causes of ascites, such as severe cardiovascular disease, renal insufficiency, extra-hepatic malignancies and non-cirrhotic portal hypertension were excluded from the study.

### Assessed parameters

Epidemiological characteristics, etiology of cirrhosis, presence of hepatocellular carcinoma (HCC), liver transplantation, varices as well as information on history of variceal bleeding were assessed from patients' medical records. Thus, information on varices was not only based on endoscopic examinations exactly at the time of the first paracententesis. Moreover, information on PPI, non-selective beta blocker (NSBB) and rifaximin intake was obtained from patients' medical records. Laboratory parameters were assessed at the first paracentesis and at the first diagnosis of SBP including platelet count, albumin, bilirubin, international normalized ratio (INR), creatinine and ascitic fluid polymorphnuclear neutrophil (PMN) count. Hepatic venous pressure gradient (HVPG) measurements were performed as described previously [22]. The model for end-stage liver disease (MELD) [23] and Child-Pugh score (CPS) [24] were calculated based on laboratory parameters and patients' medical history.

### Paracenteses, diagnosis of SBP and other infections and definition of resolution of infection

Patients were grouped according to the presence of signs or symptoms or laboratory abnormalities suggestive of or associated with infection (e.g., abdominal pain or tenderness, fever, unexplained encephalopathy, AKI, leukocytosis and variceal bleeding) and paracentesis volume: diagnostic paracentesis (paracentesis volume <5 L), diagnostic large-volume paracentesis (LVP; paracentesis volume ≥5 L), and therapeutic LVP (no clinical or laboratory evidence for infection and paracentesis volume ≥5 L).

In accordance with national guidelines [25], [26], albumin was administered in LVPs and patients received long-term prophylaxis with quinolones after SBP development. SBP was diagnosed if the ascitic PMN count was>250cells x mL^-1^ in absence of an intra-abdominal source of infection or any other explanation for an elevated PMN count [26]–[28].

In addition, patients' medical records were reviewed for hospitalizations resulting from infections other than SBP or development of systemic infections during hospitalizations due to other reasons. The standardized work up at hospital admission included laboratory blood and urine tests, as well as a chest X-ray. Systemic infections were diagnosed based on the American College of Chest Physicians (ACCP)/Society of Critical Care Medicine (SCCM) definitions for systemic inflammatory response syndrome (SIRS) and sepsis [29].

Resolution of SBP or other infections was defined by the regression of clinical or laboratory evidence for infection within 7 days after the diagnosis, as well as the absence or regression of infection-related complications such as grade 3/4 hepatic encephalopathy according to West Haven criteria [30] and acute kidney injury (AKI) within 7 days after the diagnosis of infection. AKI was defined as group C of the modified acute kidney injury classification proposed by Fagundes and co-workers [31].

### Statistics

Statistical analyses were conducted using IBM SPSS Statistics 21 (SPSS Inc., Armok, USA) and R.3.0.2 (R Core Team, R Foundation for Statistical Computing, Vienna, Austria). Continuous variables were reported as mean ±standard deviation or median (interquartile range), while categorical variables were reported as numbers (proportions) of patients with the certain characteristic. Student's t-test was used for group comparisons of continuous variables when applicable. Otherwise, Mann-Whitney U test was applied. Group comparisons of categorical variables were performed using either Pearson's chi-squared or Fisher's exact test.

A binary logistic regression model investigating the association between PPI intake and SBP at the first paracentesis adjusted for all variables (age, HCC, history of variceal bleeding, varices and MELD score) that were not comparable between PPI and no-PPI patients (Table 1) was calculated.

**Table 1:** Patient Characteristics.

Patient characteristics	All patients, n = 607	no-PPI, n = 87	PPI, n = 520	*P* value
Age, years	57.5±11.8	60.2±12.1	57.1±11.7	0.02
Sex				
Male	426 (70%)	59 (68%)	367 (71%)	0.602
Female	181 (30%)	28 (32%)	153 (29%)	
Etiology				
ALD	336 (55%)	41 (47%)	295 (57%)	0.38
Viral	113 (19%)	20 (23%)	93 (18%)	
ALD and viral	49 (8%)	9 (10%)	40 (8%)	
Other	109 (18%)	17 (20%)	92 (18%)	
HCC	129 (21%)	28 (32%)	101 (19%)	0.007
History of variceal bleeding	111 (18%)	9 (10%)	102 (20%)	0.038
Varices	443 (73%)	52 (60%)	391 (75%)	0.003
Upper-gastrointestinal bleeding	46 (8%)	6 (7%)	40 (8%)	0.795
At Hospital admission	32 (5%)	4 (5%)	28 (5%)	0.808
During hospitalization	14 (2%)	2 (2%)	12 (2%)	1
Portal hypertensive bleeding	35 (6%)	4 (5%)	31 (6%)	0.642
HVPG*, mmHg	18.7±6.5	17.3±5.8	18.8±6.6	0.273
MELD	17.5 (10.6)	15.2 (7.7)	18 (10.3)	0.037
CPS				
A	22 (4%)	5 (6%)	17 (3%)	0.361
B	281 (46%)	43 (49%)	238 (46%)	
C	304 (50%)	39 (45%)	265 (51%)	
Platelet count, G x L^−1^	117 (107)	138 (104)	117 (110)	0.17
Albumin, g x L^−1^	27.2±5.7	27.8±5.9	27.1±5.6	0.337
Bilirubin, mg x dL^−1^	3.2 (6.02)	2.43 (4.07)	3.34 (6.34)	0.046
INR	1.38 (0.58)	1.33 (0.47)	1.39 (0.59)	0.135
Creatinine, mg x dL^−1^	1.14 (0.78)	1.14 (0.64)	1.14 (0.78)	0.949
Rifaximin treatment	63 (10%)	6 (7%)	57 (11%)	0.25
NSBB treatment	245 (40%)	32 (37%)	213 (41%)	0.462
Hospitalization prior to paracentesis, days	1 (4)	1 (6)	1 (4)	0.343
Paracentesis indication				
Diagnostic paracentesis	258 (43%)	45 (52%)	213 (41%)	0.133
Diagnostic LVP	270 (44%)	30 (34%)	240 (46%)	
Therapeutic LVP	79 (13%)	12 (14%)	67 (13%)	
SBP at first paracentesis	114 (19%)	15 (17%)	99 (19%)	0.691
Systemic infection at first paracentesis	34 (6%)	1 (1%)	33 (6%)	0.072

*Information on HVPG was available in 220 patients.

Patient characteristics at the first paracentesis and comparison of patients with (PPI) and without (no-PPI) proton pump inhibitor therapy.

Abbreviations: PPI proton pump inhibitor; ALD alcoholic liver disease; HCC hepatocellular carcinoma; HVPG hepatic venous pressure gradient; MELD model for end-stage liver disease; CPS Child-Pugh score; INR international normalized ratio; NSBB non-selective beta blocker; LVP large-volume paracentesis.

**Figure 1:**
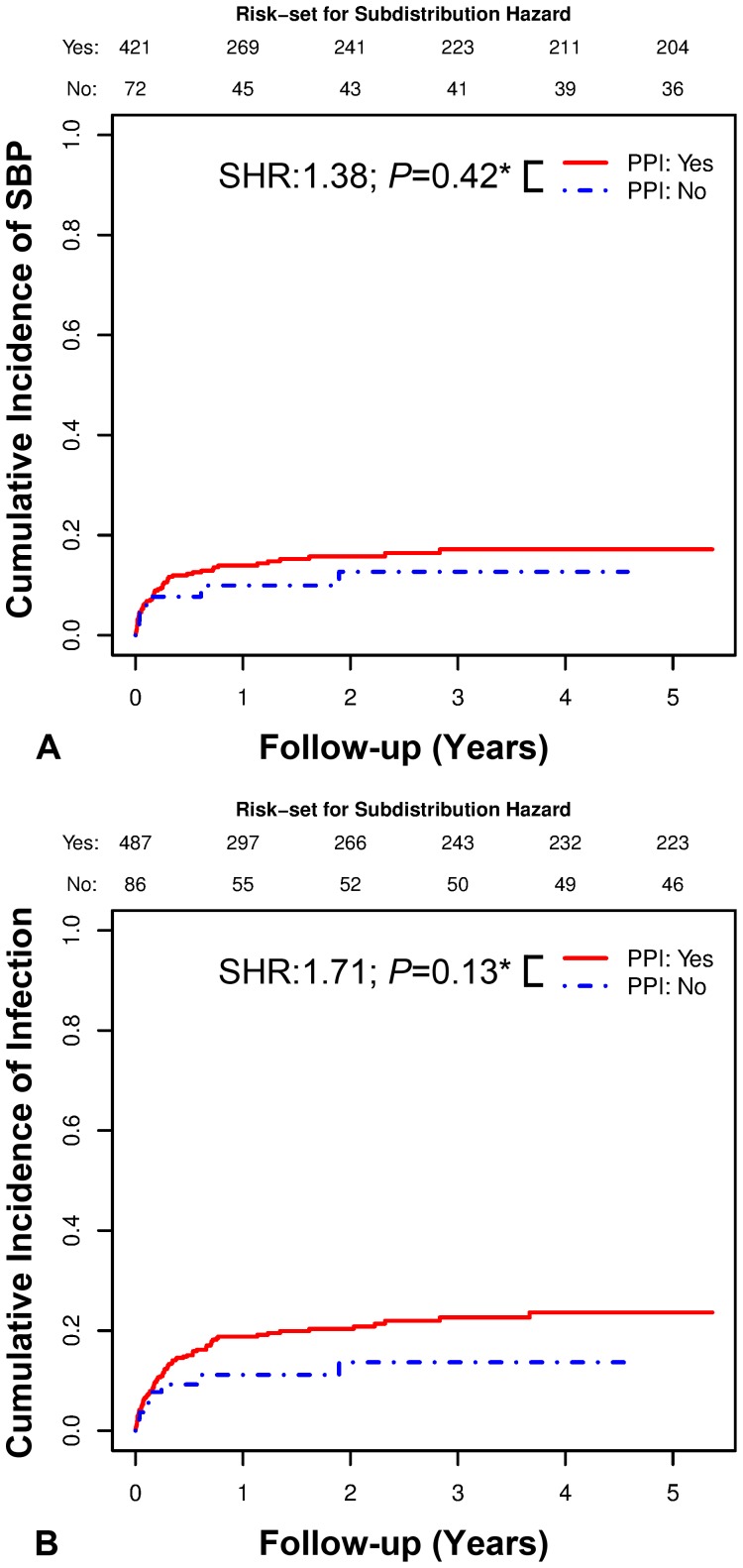
PPI Intake and Cumulative Incidence of SBP or other Infections. Impact of PPI intake on **A** cumulative incidence of SBP among patients without SBP at the first paracentesis and **B** cumulative incidence of SBP or other infections among patients without SBP or another infection at the first paracentesis. Statistics: The impact of PPI intake on the cumulative incidence of SBP or other infections was analyzed by a competing risk analysis [32] treating death as a competing risk. *In addition to PPI intake, age, HCC, history of variceal bleeding, varices and MELD score were considered covariates. Cumulative incidence functions are shown for the models investigating the incidence of SBP or other infections. Abbreviations: PPI proton pump inhibitor; SBP spontaneous bacterial peritonitis; SHR subdistribution hazard ratio; HCC hepatocellular carcinoma; MELD model for end-stage liver disease.

**Figure 2:**
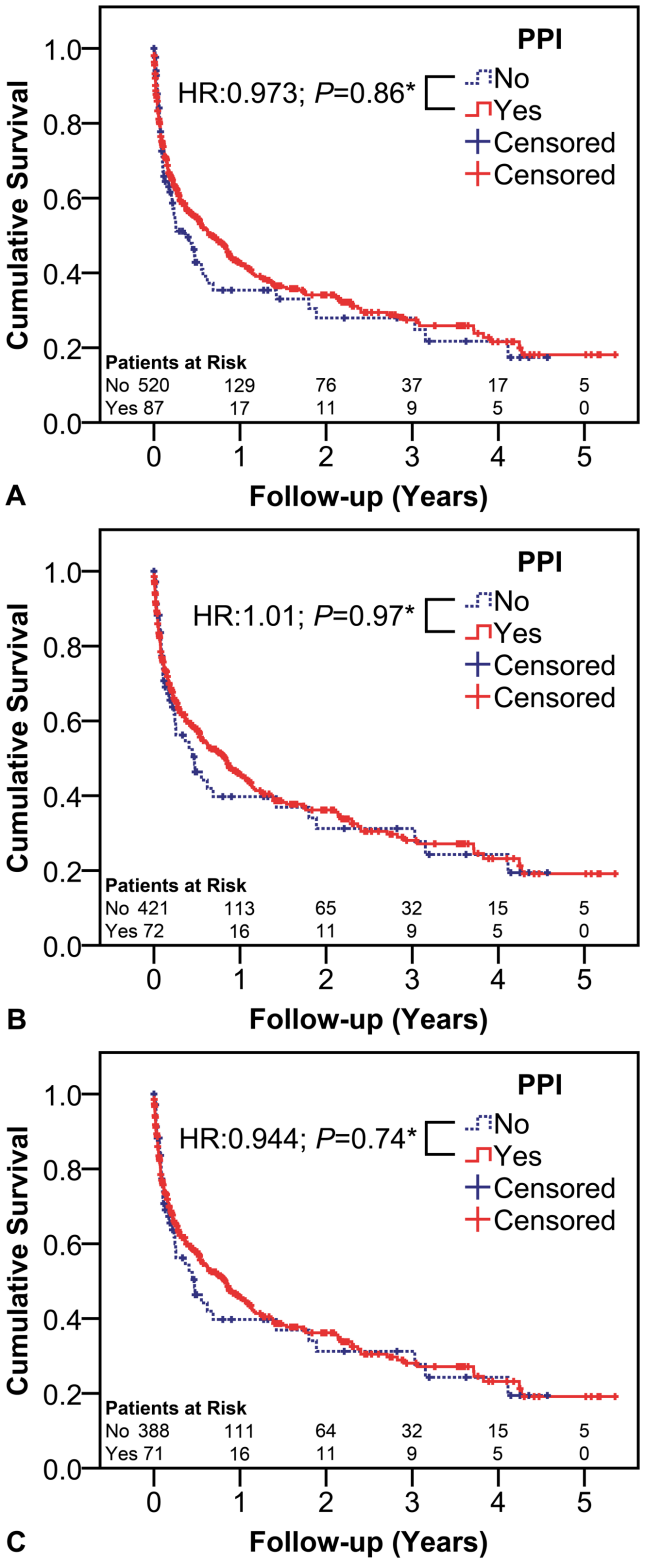
PPI Intake and Transplant-free Survival. Influence of PPI intake on transplant-free survival in **A** the overall cohort, **B** among patients without SBP and **C** among patients without SBP or others infections at the first paracentesis. Statistics: Transplant-free survival was analyzed using Cox proportional hazards models. *In addition to PPI intake, age, HCC, history of variceal bleeding, varices and MELD score were considered covariates in all of the above-mentioned models. Kaplan-Meier curves are presented for transplant-free survival models. Abbreviations: PPI proton pump inhibitor; SBP spontaneous bacterial peritonitis; HR hazard ratio; HCC hepatocellular carcinoma; MELD model for end-stage liver disease.

The impact of PPI intake on the cumulative incidence of SBP or other infections was analyzed by a competing risk analyses [32] treating death as a competing risk. Cumulative incidence functions are shown for the models investigating the incidence of SBP or other infections (Figure 1). Transplant-free survival was analyzed using Cox proportional hazards models. Patients who underwent a liver transplantation were censored on the day of surgery. Transplant-free survival time was defined as the time to liver transplantation, death or end of follow-up. Kaplan-Meier curves are presented for transplant-free survival models (Figure 2). In addition to PPI intake, age, HCC, history of variceal bleeding, varices and MELD score were considered as covariates in all of the above-mentioned models, as they were not comparable between PPI and no-PPI patients (Table 1).

All patients without SBP entered the SBP cumulative incidence model (Model 1) with their first paracentesis, while the SBP or other infections cumulative incidence model (Model 2) was restricted to patients without SBP or another infection at the first paracentesis.

Moreover, we assessed the effect of PPI intake on transplant-free survival in the overall cohort (Model 3), among patients without SBP (Model 4) and among patients without SBP or other infections at the first paracentesis (Model 5).

*P* values <0.05 were considered as statistically significant.

### Ethics

This study was conducted in accordance with the Declaration of Helsinki and approved by the local ethics committee of the Medical University of Vienna (EK Nr. 1008/2011). Due to the retrospective design of the study, the local ethics committee did not require a written informed consent from the study participants. Patient data was pseudonymized prior to statistical analysis.

## Results

### Patient characteristics at the first paracentesis (Table 1)

The majority of patients (70%) were male, with a mean age of 57.5±11.8 years. The predominant etiology of cirrhosis was alcoholic liver disease (ALD) (55%), followed by chronic viral hepatitis (19%) and the combination of ALD and chronic viral hepatitis (8%). In 18% percent of patients, other etiologies of cirrhosis were reported. Twenty-one percent of patients were diagnosed with HCC and 18% had a history of variceal bleeding, though varices were present in 73% of patients. Five percent of patients presented with upper-gastrointestinal bleeding at hospital admission, while 2% developed upper-gastrointestinal bleeding during hospitalization. Portal hypertensive bleeding at admission or during hospitalization was observed in 6% of patients. Information on HVPG was available in a subgroup of 220 patients, with a mean HVPG of 18.7±6.5 mmHg. The median MELD score was 17.5 (10.6) and the distribution of CPS stage was as follows: A: 4%, B: 46% and C: 50%. Ten percent of patients received rifaximin, while 40% of patients were administered NSBB treatment. The median duration of hospitalization prior to the paracentesis was 1 (4) day. Forty-three percent of paracenteses were diagnostic, 44% were diagnostic LVPs and 13% were therapeutic LVPs.

Among 607 cirrhotic patients with ascites, PPI intake was present in 520 (86%) of patients. At the first paracentesis, mean age was lower (PPI: 57.1±11.7 vs. no-PPI: 60.2±12.1 years; *P* = 0.02), while median MELD score was higher (PPI: 18 (10.3) vs. no-PPI: 15.2 (7.7); *P* = 0.037) among patients with PPI intake. While the proportion of patients with HCC was higher among patients without PPI intake (PPI: 19% vs. no-PPI: 32%; *P* = 0.007), history of variceal bleeding (PPI: 20% vs. no-PPI: 10%; *P* = 0.038) and varices (PPI: 75% vs. no-PPI: 60%; *P* = 0.003) were more frequently observed in the PPI group. No other statistically significant differences in patient characteristics between patients with PPI intake, and without, were observed.

Patient characteristics of the subgroups of patients without SBP and patients without SBP or other infections at the first paracentesis are shown in Table S1 and Table S2, respectively.

### Follow-up of patients

A total of 607 patients were followed for 486 person-years after their first paracentesis. Of these, 59% underwent a liver transplantation or died and 7% were lost to follow-up. The proportion of patients who were lost to follow-up was similar in the subgroups of patients with PPI intake (7%) and without (6%; *P* = 0.642).

### Impact of PPI intake on SBP prevalence at the first paracentesis and SBP incidence during follow-up

The proportion of patients with SBP at the first paracentesis was comparable between the PPI (19%) and no-PPI (17%; *P* = 0.691) group. In multivariate logistic regression analysis, neither PPI treatment (odds ratio (OR): 1.11, 95% confidence interval (95%CI): 0.602–2.061; *P* = 0.731), nor any of the other covariates including age (per 10 years, OR: 1, 95%CI: 0.99–1; *P* = 0.704), HCC (OR: 1.48, 95%CI: 0.91–2.41; *P* = 0.116), history of variceal bleeding (OR: 0.673, 95%CI: 0.376–1.205; *P* = 0.183), varices (OR: 1.54; 95%CI: 0.93–2.55; *P* = 0.093) and MELD score (per point, OR: 1.02, 95%CI: 1–1.05; *P* = 0.077) were associated with SBP prevalence.

Among patients without SBP at the first paracentesis, PPI intake was not associated with the cumulative incidence of SBP (subdistribution hazard ratio (SHR): 1.38; 95%CI: 0.63–3.01; *P* = 0.42) during follow-up when adjusting for age, HCC, history of variceal bleeding, varices and MELD score (Figure 1; Table 2). We observed a trend toward an increased cumulative incidence of SBP among patients with a history of variceal bleeding (SHR: 1.71, 95%CI: 0.96–3.05; *P* = 0.07).

**Table 2:** PPI Intake and Cumulative Incidence of SBP or other Infections.

	A Cumulative incidence of SBP n = 493, Model 1				B Cumulative incidence of SBP or systemic infection n = 459, Model 2			
Patient characteristics	SHR	95%CI		*P* value	SHR	95%CI		*P* value
		lower	upper			lower	upper	
Age, per 10 years	1	0.98	1.02	0.71	1	0.98	1.02	0.96
HCC, yes	1.35	0.74	2.46	0.33	1.01	0.61	1.68	0.97
History of variceal Bleeding, yes	1.71	0.96	3.05	0.07	1.46	0.91	2.35	0.12
Varices, yes	0.82	0.48	1.64	0.69	0.9	0.55	1.48	0.68
MELD, per point	0.99	0.96	1.02	0.4	0.98	0.96	1.01	0.18
PPI, yes	1.38	0.63	3.01	0.42	1.71	0.85	3.44	0.13

Impact of PPI intake on **A** cumulative incidence of SBP among patients without SBP at the first paracentesis and **B** cumulative incidence of SBP or other infections among patients without SBP or another infection at the first paracentesis.

Statistics: The impact of PPI intake on the cumulative incidence of SBP or other infections was analyzed by a competing risk analysis [32] treating death as a competing risk. In addition to PPI intake, age, HCC, history of variceal bleeding, varices and MELD score were considered covariates.

Abbreviations: PPI proton pump inhibitor; SBP spontaneous bacterial peritonitis; SHR subdistribution hazard ratio; HCC hepatocellular carcinoma; MELD model for end-stage liver disease.

### Influence of PPI intake on the prevalence of systemic infections at the first paracentesis and incidence of SBP or other infections during follow-up

Systemic non-SBP infections were classified as sepsis (31%), pneumonia (22%), urinary tract infections (11%), cellulitis (5%), C. difficile infection (4%) and other specific infections (11%). In 16% of patients, no specific type of infection could be identified, although they presented with clinical or laboratory evidence for systemic infection.

There was a trend toward a higher proportion of patients with systemic infections other than SBP at the first paracentesis in the PPI group (PPI: 6% vs. no-PPI: 1%; *P* = 0.072). When considering both SBP and other systemic infections as a combined event, prevalence rates at the first paracentesis were comparable between treatment groups (PPI: 25% vs. no-PPI: 18%; *P* = 0.16).

Among patients without SBP or another infection at the first paracentesis, the association between PPI intake and the cumulative incidence of the combined event (SHR: 1.71; 95%CI: 0.85–3.44; *P* = 0.13) during follow-up did not attain statistical significance, when adjusting for age, HCC, history of variceal bleeding, varices and MELD score (Figure 1; Table 2).

### Impact of PPI intake on resolution of SBP or other infections

Resolution was assessed for all SBPs and other systemic infections at the first paracentesis, as well as all incident SBPs and other systemic infections during follow-up (n = 233). The proportion of patients in which the infection was resolved was similar among patients with PPI intake (56%), and without (52%; *P* = 0.686). Moreover, we observed similar rates of infection resolution in the subgroup of patients with SBP (PPI: 60% vs. no-PPI: 50%; *P* = 0.36).

### Impact of PPI intake on transplant-free survival

The influence of PPI intake on transplant-free survival was studied in the overall cohort, among patients without SBP and among patients without SBP or other infections at the first paracentesis (Figure 2; Table 3). While higher age (per 10 years, HR: 1.38, 95%CI: 1.25–1.53, *P*<0.001), the presence of HCC (HR: 2.41, 95%CI: 1.88–3.08; *P*<0.001) and higher MELD score (per point, HR: 1.56, 95%CI: 1.45–1.68; *P*<0.001) were associated with decreased transplant-free survival in the overall cohort, no association with PPI intake (HR: 0.97, 95%CI: 0.72–1.32; *P* = 0.859) was observed.

**Table 3:** PPI Intake and Transplant-free Survival.

	ATransplant-free mortalityAll patientsn = 607, Model 3				BTransplant-free mortalityNo SBP at first paracentesis, n = 493, Model 4				CTransplant-free mortalityNo SBP or systemic infection at first paracentesisn = 459, Model 5			
Patient characteristics	HR	95%CI		*P* value	HR	95%CI		*P* value	HR	95%CI		*P* value
		lower	upper			lower	upper			lower	upper	
Age, per 10 years	1.38	1.25	1.53	<0.001	1.32	1.17	1.48	<0.001	1.33	1.18	1.51	<0.001
HCC, yes	2.41	1.88	3.08	<0.001	2.59	1.96	3.43	<0.001	2.79	2.09	3.74	<0.001
History of variceal bleeding, yes	1.01	0.77	1.34	0.923	1.17	0.86	1.59	0.322	1.14	0.82	1.57	0.433
Varices, yes	1.13	0.88	1.44	0.35	1.07	0.81	1.42	0.617	1.15	0.86	1.56	0.35
MELD, per point	1.56	1.45	1.68	<0.001	1.54	1.41	1.69	<0.001	1.51	1.37	1.66	<0.001
PPI, yes	0.97	0.72	1.32	0.859	1.01	0.72	1.42	0.971	0.94	0.67	1.33	0.742

Influence of PPI intake on transplant-free survival in **A** the overall cohort, **B** among patients without SBP and **C** among patients without SBP or others infections at the first paracentesis.

Statistics: Transplant-free survival was analyzed using Cox proportional hazards models. In addition to PPI intake, age, HCC, history of variceal bleeding, varices and MELD score were considered covariates in all of the above-mentioned models.

Abbreviations: PPI proton pump inhibitor; SBP spontaneous bacterial peritonitis; HR hazard ratio; HCC hepatocellular carcinoma; MELD model for end-stage liver disease.

## Discussion

The emergence of alarming results from recent studies [12]–[18] has initiated an intense debate on whether PPI intake has an adverse effect on the occurrence of infectious complications among patients with cirrhosis and ascites. However, in addition to their retrospective design, most previous studies display limitations, which must be considered. The majority of studies either investigated a relatively small sample size [13]–[17], [20], or had a case-control, rather than a longitudinal cohort design [13]–[17]. Moreover, some studies insufficiently controlled for potential confounding factors or did not consider death as a competing risk when investigating the impact of PPI treatment on the incidence of SBP or other infections.

Our study, although retrospective, is a longitudinal study based on a large, thoroughly documented cohort of patients with cirrhosis and ascites and applied competing risk analyses [32] treating death as a competing risk. Previous studies reporting an association between PPI intake and SBP incidence were based on cohorts with a lower proportion of patients on PPI treatment [12], [13], suggesting indications for PPI administration were followed more rigorously. In contrast, in our cohort, the particularly high prevalence of PPI intake (86%) suggests that the indications for PPI treatment were interpreted liberally in daily clinical practice. However, in the context of rather high PPI intake, we observed no association between PPI intake and SBP, as well as mortality. Although the prevalence of peptic ulcers is increased among cirrhotic patients and correlates with the severity of liver disease [33], PPI intake might have been initiated based on indications which are not sufficiently supported by evidence, such as portal hypertensive gastropathy, varices or history of variceal bleeding, abdominal pain or discomfort induced by distension of the abdomen, as well as polypharmacy [34].

In our study, the proportion of patients with HCC was higher in the no-PPI group, while the proportions of patients with varices and a history of variceal bleeding were higher in the PPI group. PPI intake was associated with higher age and MELD score, factors that were associated with lower transplant-free survival in our study. In addition, patients with a history of variceal bleeding had a numerically higher risk of SBP development during follow-up. Other potential confounding factors, such as the portal hypertensive bleeding at admission or during hospitalization, duration of hospitalization prior to paracentesis and indication for paracentesis were assessed and found to be comparable between patients with PPI intake, and without. However, the retrospective assessment of the indication for paracentesis has limitations and since the American Association for the Study of the Liver (AASLD) practice guideline for the management of adult patients with ascites due to cirrhosis [28] recommends paracentesis at the first development of ascites and at hospital admission, none of the paracenteses might have been solely therapeutic. Importantly, most previous studies did not provide sufficient information on these potential confounding factors. Thus, it cannot be excluded that the severity of the underlying liver disease and other factors, rather than PPI treatment *per se,* may predispose for infectious complications in patients with cirrhosis and ascites.

Moreover, hospitalization due to infections other than SBP or development of systemic infections during hospitalization for other reasons was assessed. Systemic infections during follow-up were not very common in our cohort of cirrhotic patients with ascites. The retrospective assessment of systemic inflammatory response syndrome (SIRS) and sepsis according to the American College of Chest Physicians (ACCP)/Society of Critical Care Medicine (SCCM) definitions [29] has inherent weaknesses, especially in patients with liver cirrhosis, in which the diagnostic capacity of SIRS criteria might already be limited [35], [36]. The retrospective assessment could have had an impact on the prevalence and incidence of systemic infections observed in our study, although there was a standardized work up at hospital admission including laboratory blood and urine tests, as well as a chest X-ray. Moreover, infections treated in an outpatient setting were not assessed. Since hospitalization is generally recommended for cirrhotic patients with ascites presenting with signs and symptoms of systemic infection, this might not have significantly affected our results. Moreover, as patients have not been prospectively followed, we cannot entirely rule out that some events were missed, especially if patients were treated outside of Vienna or in private hospitals. We observed a trend toward a higher prevalence of systemic infections other than SBP at the first paracentesis in the PPI group. However, this was an unadjusted analysis not considering the previously mentioned unfavorable baseline characteristics of the PPI group, as multivariate analysis was not feasible due to the low number of events. When considering both SBP and other systemic infections as a combined event, prevalence rates at the first paracentesis were comparable between the treatment groups. Importantly, there was no association between PPI intake and the combined event during follow-up, when adjusting for potential confounding factors.

In conclusion, we observed no association between PPIs and SBP or other infections, as well as mortality, in our large, thoroughly documented cohort of patients with cirrhosis and ascites with a particularly high prevalence of PPI intake. The severity of the underlying liver disease and other factors, rather than PPI treatment *per se* may predispose for complications in patients with cirrhosis and ascites. Nevertheless, the restriction of PPI treatment to evidence-based indications should be emphasized.

## Supporting Information

Table S1 Patient characteristics of patients without SBP at the first paracentesis and comparison of patients with (PPI) and without (no-PPI) proton pump inhibitor therapy. Abbreviations: PPI proton pump inhibitor; ALD alcoholic liver disease; HCC hepatocellular carcinoma; HVPG hepatic venous pressure gradient; MELD model for end-stage liver disease; CPS Child-Pugh score; INR international normalized ratio; NSBB non-selective beta blocker; LVP large-volume paracentesis.
(DOCX)

Table S2 Patient characteristics of patients without SBP or other infection at the first paracentesis and comparison of patients with (PPI) and without (no-PPI) proton pump inhibitor therapy. Abbreviations: PPI proton pump inhibitor; ALD alcoholic liver disease; HCC hepatocellular carcinoma; HVPG hepatic venous pressure gradient; MELD model for end-stage liver disease; CPS Child-Pugh score; INR international normalized ratio; NSBB nonselective beta blocker; LVP large-volume paracentesis.
(DOCX)
