# Supplementary material for: Proton Pump Inhibitor Intake neither Predisposes to Spontaneous Bacterial Peritonitis or Other Infections nor Increases Mortality in Patients with Cirrhosis and Ascites
Source: PLoS One. 2014 Nov 4;9(11):e110503. doi: 10.1371/journal.pone.0110503 (PMC4219684; doi:10.1371/journal.pone.0110503)
Supplement: Table S1 — Patient characteristics of patients without SBP at the first paracentesis and comparison of patients with (PPI) and without (no-PPI) proton pump inhibitor therapy. Abbreviations: PPI proton pump inhibitor; ALD alcoholic liver disease; HCC hepatocellular carcinoma; HVPG hepatic venous pressure gradient; MELD model for end-stage liver disease; CPS Child-Pugh score; INR international normalized ratio; NSBB non-selective beta blocker; LVP large-volume paracentesis. (DOCX) [file pone.0110503.s001.docx]

**Table S1**

| Patient characteristics | | All patients,  n=493 | no-PPI,  n=72 | PPI,  n=421 | *P* value |
| --- | --- | --- | --- | --- | --- |
| Age, years | | 58.2 ±11.7 | 60.3 ±12.5 | 57.1±11.5 | 0.033 |
| Sex | | | | | |
|  | Male | 347 (70%) | 50 (69%) | 297 (71%) | 0.85 |
|  | Female | 146 (30%) | 22 (31%) | 124 (29%) |  |
| Etiology | | | | | |
|  | ALD | 283 (57%) | 34 (47%) | 249 (59%) | 0.224 |
|  | Viral | 84 (17%) | 17 (24%) | 67 (16%) |  |
|  | ALD and viral | 42 (9%) | 8 (11%) | 34 (8%) |  |
|  | Other | 84 (17%) | 13 (18%) | 71 (17%) |  |
| HCC | | 100 (20%) | 23 (32%) | 77 (18%) | 0.008 |
| History of variceal bleeding | | 94 (19%) | 7 (10%) | 87 (21%) | 0.029 |
| Varices | | 354 (72%) | 43 (60%) | 311 (74%) | 0.014 |
| Upper-gastrointestinal bleeding | | 38 (8%) | 4 (6%) | 34 (8%) | 0.459 |
|  | At Hospital admission | 27 (5%) | 3 (4%) | 24 (6%) | 0.782 |
|  | During hospitalization | 11 (2%) | 1 (1%) | 10 (2%) | 0.713 |
|  | Portal hypertensive bleeding | 30 (6%) | 3 (4%) | 27 (6%) | 0.6 |
| HVPG*, mmHg | | 18.9 ±6.4 | 17.9 ±6.2 | 19.1 ±6.5 | 0.472 |
| MELD | | 17.3 (9.43) | 15.4 (7.7) | 17.8 (9.44) | 0.057 |
| CPS | | | | | |
|  | A | 18 (4%) | 4 (6%) | 14 (3%) | 0.246 |
|  | B | 235 (48%) | 39 (54%) | 196 (47%) |  |
|  | C | 240 (49%) | 29 (40%) | 211 (50%) |  |
| Platelet count, G x L^-1^ | | 116 (101) | 129 (94) | 115 (104) | 0.404 |
| Albumin, g x L^-1^ | | 27.2 ±5.5 | 27.1 ±5.6 | 27.2 ±5.5 | 0.966 |
| Bilirubin, mg x dL^-1^ | | 3.28 (5.81) | 2.43 (5.25) | 3.33 (5.99) | 0.193 |
| INR | | 1.39 (0.55) | 1.33 (0.33) | 1.39 (0.58) | 0.066 |
| Creatinine, mg x dL^-1^ | | 1.1 (0.74) | 1.11 (0.6) | 1.1 (0.78) | 0.629 |
| Rifaximin treatment | | 56 (11%) | 6 (8%) | 50 (12%) | 0.381 |
| NSBB treatment | | 187 (38%) | 26 (36%) | 161 (38%) | 0.731 |
| Hospitalization prior to paracentesis, days | | 1 (5) | 1 (5) | 1 (4) | 0.682 |
| Paracentesis indication | | | | | |
|  | Diagnostic paracentesis | 192 (39%) | 35 (49%) | 157 (37%) | 0.104 |
|  | Diagnostic LVP | 227 (46%) | 25 (35%) | 202 (48%) |  |
|  | Therapeutic LVP | 74 (15%) | 12 (17%) | 62 (15%) |  |
| Systemic infection at first paracentesis | | 34 (7%) | 1 (1%) | 33 (8%) | 0.072 |

* Information on HVPG was available in 171 patients.
