# Supplementary material for: Proton Pump Inhibitor Intake neither Predisposes to Spontaneous Bacterial Peritonitis or Other Infections nor Increases Mortality in Patients with Cirrhosis and Ascites
Source: PLoS One. 2014 Nov 4;9(11):e110503. doi: 10.1371/journal.pone.0110503 (PMC4219684; doi:10.1371/journal.pone.0110503)
Supplement: Table S2 — Patient characteristics of patients without SBP or other infection at the first paracentesis and comparison of patients with (PPI) and without (no-PPI) proton pump inhibitor therapy. Abbreviations: PPI proton pump inhibitor; ALD alcoholic liver disease; HCC hepatocellular carcinoma; HVPG hepatic venous pressure gradient; MELD model for end-stage liver disease; CPS Child-Pugh score; INR international normalized ratio; NSBB nonselective beta blocker; LVP large-volume paracentesis. (DOCX) [file pone.0110503.s002.docx]

**Table S2**

| Patient characteristics | | All patients,  n=459 | no-PPI,  n=71 | PPI,  n=388 | *P* value |
| --- | --- | --- | --- | --- | --- |
| Age, years | | 57.5 ±11.6 | 60.2 ±12.7 | 57 ±11.3 | 0.03 |
| Sex | | | | | |
|  | Male | 322 (70%) | 49 (69%) | 273 (70%) | 0.82 |
|  | Female | 137 (30%) | 22 (31%) | 115 (30%) |  |
| Etiology | | | | | |
|  | ALD | 266 (58%) | 34 (48%) | 232 (60%) | 0.297 |
|  | Viral | 83 (18%) | 17 (24%) | 66 (17%) |  |
|  | ALD and viral | 38 (8%) | 7 (10%) | 31 (8%) |  |
|  | Other | 72 (16%) | 13 (18%) | 59 (15%) |  |
| HCC | | 95 (21%) | 23 (32%) | 72 (19%) | 0.004 |
| History of variceal bleeding | | 87 (19%) | 6 (8%) | 81 (21%) | 0.014 |
| Varices | | 332 (72%) | 42 (59%) | 290 (75%) | 0.007 |
| Upper-gastrointestinal bleeding | | 37 (8%) | 4 (6%) | 33 (9%) | 0.414 |
|  | At Hospital admission | 26 (6%) | 3 (4%) | 23 (6%) | 0.606 |
|  | During hospitalization | 11 (2%) | 1 (1%) | 10 (3%) | 0.706 |
|  | Portal hypertensive bleeding | 29 (6%) | 3 (4%) | 26 (7%) | 0.598 |
| HVPG*, mmHg | | 19 ±6.4 | 17.9 ±6.2 | 19.1 ±6.5 | 0.455 |
| MELD | | 17 (9.3) | 15.4 (7.7) | 17.2 (9.3) | 0.137 |
| CPS | | | | | |
|  | A | 17 (6%) | 4 (6%) | 13 (3%) | 0.382 |
|  | B | 225 (49%) | 38 (54%) | 187 (48%) |  |
|  | C | 217 (47%) | 29 (41%) | 188 (48%) |  |
| Platelet count, G x L^-1^ | | 117 (100) | 129 (94) | 116 (100) | 0.462 |
| Albumin, g x L^-1^ | | 27.3 ±5.5 | 27.1 ±5.6 | 27.4 ±5.5 | 0.773 |
| Bilirubin, mg x dL^-1^ | | 3.18 (5.43) | 2.43 (5.25) | 3.27 (5.52) | 0.277 |
| INR | | 1.38 (0.53) | 1.33 (0.33) | 1.38 (0.55) | 0.133 |
| Creatinine, mg x dL^-1^ | | 1.09 (0.67) | 1.11 (0.59) | 1.09 (0.72) | 0.389 |
| Rifaximin treatment | | 56 (11%) | 6 (8%) | 50 (12%) | 0.381 |
| NSBB treatment | | 187 (38%) | 26 (36%) | 161 (38%) | 0.731 |
| Hospitalization prior to paracentesis, days | | 1 (5) | 1 (5) | 1 (4) | 0.723 |
| Paracentesis indication | | | | | |
|  | Diagnostic paracentesis | 173 (38%) | 34 (48%) | 139 (36%) | 0.098 |
|  | Diagnostic LVP | 73 (16%) | 12 (17%) | 61 (16%) |  |
|  | Therapeutic LVP | 213 (46%) | 25 (35%) | 188 (48%) |  |

* Information on HVPG was available in 166 patients.
